# The rewarming benefit of anterior torso heat pad application in mildly hypothermic conscious adult trauma patients remains inconclusive

**DOI:** 10.1186/1757-7241-20-17

**Published:** 2012-03-04

**Authors:** Joseph Yuk Sang Ting

**Affiliations:** 1 Retrieval and Clinical Coordination Physician, Careflight Medical Services, 160 Robina Town Centre Drive, Robina QLD 4226, Australia

**Keywords:** Hypothermia, Trauma, Rewarming, Heat pad

## Abstract

The rewarming benefit of anterior torso heat pad application in mildly hypothermic conscious adult trauma patients remains inconclusive in this randomized comparative clinical trial. There was no between-group rewarming gain in ear canal temperature when an anterior torso chemical heat pad was compared with blankets. Patient awareness, and favorable perception of, being administered the active intervention (heat pad) could explain the significant improvement in patient-rated cold discomfort discerned with the heat pad. In the context of marginal demonstrated benefit, it would have been informative to ascertain adverse effects related to the heat pad, including burn injury to the chest wall.

## Letter to Editor

In their randomized clinical trial of managing hypothermia in trauma, Lundgren at al [1] attempt to demonstrate the external warming capability of a chemical heat pad applied to the upper anterior torso of lucid mildly hypothermic trauma patients. They found only a 0.9 and 0.8°C increment in mean ear canal temperature in the passive and heat pad warming groups respectively. Assuming non-biased temperature measurement error (+/-0.2°C), this finding implies the heat pad to have conferred no additional benefit in terms of heat gain achieved with just blankets used in the comparison arm.

Improvements in heart rate, systolic blood pressure and respiratory rate seem to not be clinically consequential; the heat pad was associated with - 4 beats/min for heart rate compared with -1 beat/min in patients that were allocated blankets. The favourably biased view that a patient holds of any active treatment in a non-blinded intervention trial [2] could substantially explain the significant improvement in patient-rated cold discomfort discerned with the heat pad. This is supported by the lack of between-group difference in core temperature gain during patient transport, which was implied to be the primary outcome. The preliminary report of heat pad use by Lundgren et al demonstrated an esophageal temperature gain of just 1°C over a relatively prolonged 80 minutes in healthy adult volunteers who had their shivering pharmacologically suppressed [3]. A posterior heat pad used in concert with the anterior pad [3] could have accelerated rewarming in this clinical trial-it is unclear why the former was not used.

A heat pad weighing 1400 grams [3] could theoretically impose detrimental restriction on a spontaneously breathing or mechanically ventilated trauma patient with significant chest injury. A non see-through anterior pad could interfere with visual assessment of chest excursion during transport. In his volunteer study, Lundgren P et al [3] refer to risk of burn injury in the first 30 minutes after heat pad application which could be mitigated by the placement of a towel between pad and skin. In a trial where anterior heat pad was found to not confer additional rewarming benefit compared with just blanket coverage in mildly hypothermic conscious trauma patients, it would have been informative to ascertain adverse effects or events related to the device, including burn injury to the anterior chest wall.

Although spontaneous hypothermia in conjunction with acidosis and coagulopathy is harmful in severe trauma [4], there is preliminary evidence of improved outcomes with cooling to 32-34°C in severe isolated traumatic brain injury [5], severe spinal cord injury [6], and penetrating trauma associated with peri-arrest [7]. Lundgren et al [1] could have advised caution with augmented or accelerated rewarming in these at-risk trauma subgroups.

## Abbreviations

°C: degrees centigrade

## Competing interests

The author declares that they have no competing interests.
